# Plasma DPP4 activity is associated with no-reflow and major bleeding events in Chinese PCI-treated STEMI patients

**DOI:** 10.1038/srep39412

**Published:** 2016-12-21

**Authors:** Jing Wei Li, Yun Dai Chen, Wei Ren Chen, Jing Jing, Jie Liu, Yong Qiang Yang

**Affiliations:** 1 Department of Cardiology, People’s Liberation Army General Hospital, Beijing, China; 2 Department of Cardiology, Xinqiao Hospital, Third Military Medical University, Chongqing, China

## Abstract

Dipeptidyl peptidase-4 (DPP4) is an important regulator of incretins and inflammation, and it is involved in the pathophysiological process of myocardial infarction (MI). This study investigated the role of plasma DPP4 activity (DPP4a) in patients with ST-segment elevation myocardial infarction (STEMI) who had undergone percutaneous coronary intervention (PCI). We recruited 747 consecutive PCI-treated STEMI patients from a tertiary referral center from January 2014 to October 2015. The outcomes of interest were the rates of no-reflow, in-hospital major adverse cardiac or cerebrovascular events (iMACCE), in-hospital complications (IHC) and in-hospital major bleeding. The DPP4a was lower in STEMI patients compared with the controls (p < 0.0001). Multivariate logistic-regression analyses (adjusted for confounding variables) showed that a 1 U/L increase in DPP4a was associated with an increased rate of no-reflow events (odds ratio [OR]: 1.07; 95% CI: 1.02–1.11; p < 0.01) and a decreased rate of major bleeding events (OR: 0.90; 95% CI: 0.82–0.98; p = 0.02). There were no associations between DPP4a and either iMACCE or IHC. In conclusions, high levels of DPP4a are independently associated with an increased rate of no-reflow events and a decreased rate of major bleeding events in PCT-treated STEMI patients.

ST-segment elevation myocardial infarction (STEMI) is an acute manifestation of coronary heart disease, and it is a frequent cause of death^1^. A better understanding of the risk factors and pathogenic mechanisms underlying STEMI may help to improve the patients’ prognosis and quality of life.

Dipeptidyl peptidase 4 (DPP4) is an exopeptidase that is expressed on the surface of a diverse range of cells. It is a protease that cleaves off amino-terminal dipeptides that have L-proline, L-alanine or serine in the penultimate position^2^. As a cell surface protein, DPP4 is involved in regulation of the immune system, signal transduction and apoptosis^3^. A soluble form of DPP4 circulates in the plasma. Soluble DPP4 comes from clearance of membrane-type DPP4 or it is secreted by cells (such as endothelial cells). Soluble DPP4 also has membrane-type DPP4 enzymatic activity^4^. The levels of plasma DPP4 activity (DPP4a) are elevated in several diseases, including type 2 diabetes mellitus (T2DM), obesity^5^, atherosclerosis^6^, and osteoporosis^7^.

Research has shown that treatment with DPP4 inhibiters before cardiac ischemia-reperfusion (I/R) injuries leads to improved survival rates and better heart function in rats, which is partly due to the activation of the phospho-Ak mouse strain thymoma (pAkt), phospho-glycogen synthase kinase 3 (pGSK3), and atrial natriuretic peptide (ANP) pathways^8^. In addition, the inhibition of DPP4 can alleviate atherosclerosis^9^ and heart failure^10^. Accordingly, one could hypothesize that high levels of DPP4a may worsen myocardial I/R injuries, causing poorer cardiovascular outcomes. However, to the best of our knowledge, no study has evaluated whether DPP4a is associated with adverse clinical outcomes in STEMI patients. The aim of this study was to investigate whether plasma DPP4a is associated with adverse in-hospital cardiovascular events in these patients.

## Results

The study included 747 patients with STEMI. Most of the subjects were men (82.7%) and the mean age was 57.8 years. Among the included patients, 165 had diabetes; 123 of these diabetic patients were on oral hypoglycemic agents and 114 were using insulin. The median levels of hemoglobin A1c (HbA1c) in the diabetic patients was 7.0% (inter-quartile range, 6.3–8.1%).

**Figure 1:**
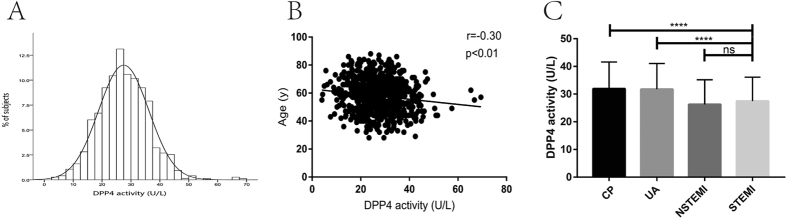
Characteristics of DPP4a at baseline in STEMI patients. (**A**) DPP4a was normally distributed. (**B**) DPP4a was negatively associated with age. (**C**) DPP4a was lower in the STEMI group compared with the CP and UA control groups. ****p < 0.0001 CP, chest pains; DPP4, dipeptidyl peptidase-4; ns, non-significant; NSTEMI, non-ST-segment elevation myocardial infarction; STEMI, ST-segment elevation myocardial infarction; UA, unstable angina.

The levels of DPP4a in the participants were normally distributed (Fig. 1A), and DPP4a was negatively associated with age (Pearson’s r = −0.30, p < 0.01; Fig. 1B) but not with fasting plasma glucose (Pearson’s r = −0.05, p = 0.20), and there was no significant difference between male and female participants (p = 0.80). The DPP4a in the STEMI patients was significantly lower than that of the chest pain (CP) and unstable angina (UA) controls, but it was not significantly different to that of the non-STEMI (NSTEMI) controls (STEMI patients: 27.49 ± 0.31 U/L, n = 747; CP controls: 31.96 ± 0.83 U/L, n = 134, p < 0.0001; UA controls: 31.76  ± 0.67 U/L, n = 190, p < 0.0001; NSTEMI controls: 26.26 ± 0.74 U/L, n = 146, p = 0.12; Fig. 1C).

The STEMI patients were divided into quartiles according to their DPP4a measurements. The individuals in the highest quartile were younger and less likely to have diabetes and had higher levels of total and LDL cholesterol and GGT, and lower level of fibrinogen (Tables 1 and 2).

The analysis of correlation demonstrated that DPP4a was positively associated with total, LDL and HDL cholesterol, thrombin time, peak CK-MB, peak myoglobin, and GGT in STEMI patients, while it was negatively associated with fibrinogen and creatinine (all p < 0.05) (Table 2). However, in the NSTEMI control group, DPP4a was not associated with fibrinogen, peak CK-MB, peak myoglobin, creatinine, thrombin time, or GGT (Supp. Table 1). The multiple linear regression analysis of the STEMI patient data showed that age, fibrinogen, peak CK-MB, and GGT (all p < 0.05) were independently associated with DPP4a in STEMI patients (Table 3).

The respective prevalences of no-reflow events, iMACCE, IHC, and in-hospital major bleeding events were 6.0%, 0.7%, 12.2%, and 2.1%, respectively. In the unadjusted analysis, the analysis that adjusted for demographic factors and the fully adjusted analysis, a higher level of DPP4a was associated with greater odds of no-reflow events (adjusted OR [aOR] 1.07 [1.02–1.11] per 1 U/L DPP4a; p < 0.01) and a lower odds of major bleeding events (aOR, 0.90 [0.82–0.98] per 1 U/L DPP4a; p = 0.02). However, DPP4a was neither significantly associated with iMACCE or IHC (Table 4) nor with any of their components. The following aORs per 1 U/L DPP4a are the aORs for each component after adjusting for age and sex: in-hospital death: 1.24 [0.99–1.56], p = 0.06; nonfatal MI: 0.95 [0.84–1.06], p = 0.34; stroke: none; acute heart failure: 1.00 [0.96–1.04], p = 0.94; atrial fibrillation: 1.00 [0.92–1.09], p = 0.99; CP: 1.02 [0.98–1.06], p = 0.45; complete atrioventricular block: 0.97 [0.88–1.07], p = 0.56; cerebrovascular disease: none; and ventricular fibrillation or ventricular tachycardia: 1.03 [0.98–1.09], p = 0.30). In the NSTEMI control group, after adjusting for age and sex, a 1 U/L increase in DPP4a was not associated with no-reflow events (aOR 1.00 [0.93–1.07], p = 0.98), iMACCE (aOR 1.33 [0.78–2.27], p = 0.30), IHC (aOR 1.18 [0.95–1.46], p = 0.13) or major bleeding events (aOR 1.05 [0.98–1.12], p = 0.17).

## Discussion

We showed that DPP4a was lower in the STEMI patients than in the CP and UA controls. Moreover, in the STEMI patients, DPP4a was positively associated with no-reflow events and negatively associated with major bleeding events. Adjusting for baseline variables did not affect these results.

A previous study reported that coronary artery disease (CAD) patients with myocardial infarction (MI) have a decreased concentration of plasma DPP4 than CAD patients without MI^11^, and a second study reported that DPP4a decreased over the 4 months after patients experienced MI^12^. However, circulating DPP4 can become inactive in certain conditions and, in these conditions, the concentration of plasma DPP4 is no longer associated with DPP4a. We showed that DPP4a also decreased after MI. Our results indicated that elevated DPP4a could be harmful to the heart and blood vessels. This concurs with previous research, that found that DPP4a increased in patients with heart failure and it was associated with poorer heart function compared with healthy subjects^13^. In addition, research has shown that higher levels of DPP4a are associated with left ventricular systolic and diastolic dysfunction^14^ and larger carotid artery intima-media thickness^6^ in T2DM patients. These results are consistent with research on animal models, in which DPP4 inhibitors were shown to improve functional recovery, decrease passive left ventricular compliance, increase endothelial cell density and decrease cardiomyocyte hypertrophy after I/R injury^4^.

However, three large randomized clinical trials failed to show that DPP4 inhibitors can decrease cardiovascular events, and one of them indicated an unexpected increase in the risk of heart failure in T2DM patients^15^. Moreover, a meta-analysis showed that DPP4 inhibitors do not decrease the risk of all-cause and cardiovascular mortality^16^. Our study also found that DPP4a was not associated with either iMACCE or IHC. These results suggest that DPP4 may play several roles in humans, which could lead to it having a neutral effect on cardiovascular events.

The occurrence of post-PCI no-reflow attenuates the benefit of reperfusion therapy and it is associated with poor clinical outcomes^17^. The pathophysiology of no-reflow is complex, as it involves cell edema, leukocyte infiltration, vasoconstriction, inflammation, and distal embolization^18^. DPP4 may affect no-reflow in both GLP-1-dependent and independent ways.

GLP-1 is an incretin that was originally found to regulate plasma glucose levels. It is rapidly inactivated by DPP4, so it has a short half-life (less than 2 min)^19^. The receptor for GLP-1 (GLP-1R) is expressed on cardiomyocytes, endothelial cells, and coronary smooth muscle cells^20^. In humans and in animal models, GLP-1R stimulation has beneficial effects on cardiac contractility, blood pressure and cardiac output, independent of its hypoglycemic effect^21^. Research has shown that GLP-1 increases the expression of p38 mitogen-activated protein kinases (MAPK), nitric oxide (NO) and glucose transporter 1 (GLUT1) and thus it has been shown to increase myocardial glucose uptake in an experimental heart I/R model^22^. Our research team previously found that GLP-1 is able to protect stem cells from heart I/R injuries^23^,^24^, and liraglutide (a GLP1 analog) can prevent no-reflow and improve heart function in STEMI patients^25^,^26^.

However, DPP4 may also act in a GLP-1R independent way. DPP4 is widely expressed on inflammatory cells and it can upregulate T-cell activation. The addition of soluble recombinant DPP4 promotes the recall antigen-induced proliferation of peripheral blood lymphocytes, while soluble mutant (inactive) DPP4 does not have this effect^27^.

We found that higher levels of DPP4a are negatively associated with major bleeding events; in fact, there were no major bleeding events in the highest DPP4a quartile. The fact that circulating DPP4a is negatively correlated with major bleeding events and positively correlated with no-reflow events indicates that DPP4a may be associated with an enhanced pro-coagulation state. A previous study found that, after AMI, DPP4 activity on the endothelial cell membranes of intramyocardial blood vessels is significantly decreased, and the loss of membrane-bound DPP4 causes the endothelial cells to change from a normal state to an enhanced pro-coagulation state^28^. It is hypothesized that soluble DPP4 is produced from membrane-bound DPP4 when it is lost from vascular endothelial cells. Thus, higher levels of DPP4a in the plasma of STEMI patients is associated with an enhanced pro-coagulation state. This hypothesis is based on the fact that soluble DPP4 could be cleaved from the membrane of human adipocytes, smooth muscle cells and liver cells by the involvement of matrix metalloproteases, a phenonemnon called “sheddases”^29^,^30^. Interestingly, we also found that DPP4a is negatively correlated with fibrinogen in STEMI patients. The decreased fibrinogen is usually associated with decreased coagulation^31^ but not with post-PCI bleeding^32^. It has been reported that the membrane-bound and soluble forms of DPP4 may have different levels of efficiency regarding their ability to cleave substrates^33^,^34^. As fibrin is a DPP4 substrate^35^, the soluble form of DPP4 may truncate fibrin more easily than the membrane-bound form, leading to a negative correlation between DPP4a and fibrinogen. Further basic research is needed to explore these issues.

## Methods

### Enrollment

The People’s Liberation Army General Hospital (PLAGH) is a large national tertiary referral center in Beijing, China. Between January 2014 and October 2015, we recruited consecutive Chinese patients with STEMI aged from 28 to 88 years who had undergone PCI at PLAGH. STEMI was defined as having typical chest pains for >30 min and elevated levels of troponin T, together with a clear ST-segment elevation of >0.1 mm in at least two contiguous electrocardiographic leads. We excluded those who were unsuitable for PCI according to the current guidelines, which included those who needed coronary artery bypass grafting (CABG), those who could be treated with thrombolytic therapy alone, those with significant hepatic or renal dysfunction and those with severe infections^36^,^37^.

Blood samples were collected at admission, and the plasma was frozen at −80 °C until further analysis. Initially, we recruited 851 patients and subsequently excluded: patients with cancer (n = 27; as cancer can affect both DPP4a and the outcomes of interest), patients who did not provide blood samples (n = 32), and patients who were taking a DPP4 inhibitor (n = 28) or a glucagon-like peptide-1 (GLP-1) analogue (n = 17). In total, 747 STEMI patients were included.

In addition, three sets of patients who attended the PLAGH during the study recruitment period were recruited for three control groups: the chest pains (CP) control group, the unstable angina (UA) control group and the non-ST segment elevation myocardial infarction (NSTEMI) control group. For the CP control group, we recruited 134 patients who had come to the hospital with CP but whose angiographic results and markers of myocardial necrosis were normal. For the UA control group, 190 patients with UA who had undergone PCI were recruited. Lastly, 146 patients with NSTEMI who had undergone PCI were recruited for the NSTEMI control group. The diagnoses of UA and NSTEMI were based on current guidelines^38^.

The study protocol was prepared in accordance with the Helsinki Declaration and it was approved by the ethics committee of the PLAGH and the Beijing Ethics Association. All the participants gave written consent to take part in the study. The PCI procedures were performed according to the current standard guidelines^36^. Each patient was required to take aspirin indefinitely after the procedure and to take clopidogrel for at least 12 months. The trial was registered with ClinicalTrials.gov (registration number: NCT02849691).

### Variable definitions

We collected clinical data on each patient, including details of their PCI procedure, in-hospital outcomes and plasma factors. In addition, the Thrombolysis In Myocardial Infarction (TIMI) and the Myocardial Blush Grade (MBG) scoring systems were used to evaluate the anterograde flow in the infarct-related coronary artery^39^,^40^. Angiographic no-reflow was defined as a TIMI grade of  <3 with a MBG of 0–1^41^.

Other outcomes of interest included in-hospital major adverse cardiac or cerebrovascular events (iMACCE), which is a composite events based on death, nonfatal MIs and strokes, and in-hospital complications (IHC), which comprised acute heart failure, atrial fibrillation, chest pain, re-acute MI, complete atrioventricular block, cerebrovascular disease, ventricular fibrillation and ventricular tachycardia. In addition, we collected data on in-hospital major bleeding events. This was defined as having an decrease in hemoglobin levels (from baseline to nadir) of ≥4 g/dL, an intracranial hemorrhage, a retroperitoneal hemorrhage, a red blood cell transfusion along with a baseline hemoglobin level of ≥9.0 g/dL, or a red blood cell transfusion along with a witnessed bleeding event and a baseline hemoglobin level of < 9.0 g/dl^42^,^43^. Hypertension was defined as a blood pressure of ≥140/90 mmHg or the use of an antihypertensive agent. Diabetes was defined as a fasting plasma glucose level of ≥7 mmol/L, a 75-g oral glucose tolerance test that resulted in a 2-h plasma glucose level of ≥11.1 mmol/L, or the use of anti-diabetic agents.

### Biochemical measurements

Plasma DPP4a was determined by measuring the quantity of p-nitroaniline (pNa) cleaved by DPP4 from the substrate H-Gly–Pro-pNa (L1880, Bachem, Switzerland), with slight changes^44^. In brief, for each participant, 5-μl blood sample was added to 150-μl 50 mM tris-HCl that contained 1 mM H-Gly–Pro-pNa. The absorbance at 405 nm was measured at 0 min and 60 min. The activity was expressed in units of U/L, with 1 U representing the production of 1 mmol of pNa every minute at 37 °C. We also obtained additional biochemical blood measurements including levels of fast plasma glucose, HbA1c, total cholesterol, low-density lipoprotein (LDL) cholesterol, high-density lipoprotein (HDL) cholesterol, triglycerides, pro-brain natriuretic peptide (pro-BNP), creatinine, MB isoenzyme of creatine kinase (CK-MB), thrombin time, prothrombin time, activated partial thromboplastin time (APTT), fibrinogen, and gamma-glutamyltransferase (GGT) using standard clinical analytical methods (AU5800, Beckman, USA; CA-7000, Sysmex, Japan).

### Statistical analysis

The PP and QQ plots were used to test the normality of the data. Where the data associated with continuous variables were normally distributed, the results were presented as means ± SDs. Some of the plasma factors were not normally distributed, and these results were presented as medians (inter-quartile range). The data associated with dichotomous variables were presented as frequencies (with percentages). We divided the participants into four categories according to the DPP4a quartile cut-off values (which were 21.54 U/L, 27.15 U/L and 32.96 U/L) and we used these categories in the subsequent analyses.

The clinical and biochemical characteristics that were normally distributed were compared using analyses of variance (ANOVAs), while the non-normal variables were compared using Kruskal–Wallis tests and the dichotomous variables were compared using chi-square tests. Spearman’s rank correlation coefficient was used to analyze the correlation between DPP4a and plasma factors that were not normally distributed, and Pearson’s correlation coefficient was used if they were normally distributed.

In addition, the associations between DPP4a and the plasma or clinical factors were assessed using a multivariate linear regression model with a forward selection approach. The variables considered for inclusion in the model included age, gender, body mass index (BMI), total cholesterol, LDL cholesterol, HDL cholesterol, triglycerides, creatinine, peak CK-MB, peak myoglobin, peak cardiac troponin T (cTNT), fasting plasma glucose, thrombin time, prothrombin time, APTT, fibrinogen, D-dimers, GGT, pro-BNP, history of hypertension, previous MI, smoking status, use of an angiotensin-converting-enzyme inhibitor (ACEI) or an angiotensin receptor blocker (ARB), statin use, aspirin use, β-blocker use, and aldosterone antagonist use. The normality of the residuals was tested by plotting a histogram and a P-P plot of the regression standardized residuals, which indicated that the residuals had a normal distribution (data not shown). The multicollinearity of the variables used in the linear regression model was tested by calculating the tolerance and the variance inflation factor (VIF). To avoid multicollinearity, we included GGT in the multiple linear regression but not aspartate transaminase (AST) or alanine transaminase (ALT). The tolerance values of age, GGT, peak CK-MB and fibrinogen were greater than 0.94 and VIF values were less than 1.07, which indicates nonsignificant multicollinearity.

Subsequently, multivariate logistic regression models were estimated to identify potential independent relationships between DPP4a and no-reflow events, iMACCE, IHC, and major bleeding events. A forward selection approach was used to select the variables to include in the models. The variables adjusted in the regression models were those that were found to be statistically significant during forward-selection approach or biologically relevant (Model 3), which included adjusting for age, sex, BMI, creatinine, AST, total cholesterol, fibrinogen, peak CK-MB, pro-BNP, fasting plasma glucose, history of hypertension, previous MI, smoking status, ACEI/ARB use, and β-blocker use. In each of the logistic regression models, DPP4a was analyzed in quartiles and as a continuous variable. The odds ratios (ORs) and 95% CIs were reported. Hosmer-Lemeshow goodness-of-fit statistic test were used in all logistic regression analysis, with all P > 0.1. All the statistical analyses were performed using SPSS 13.0 software (SPSS, IL, USA). A two-sided p value of < 0.05 was considered to represent statistical significance.

## Additional Information

**How to cite this article**: Li, J. W. *et al*. Plasma DPP4 activity is associated with no-reflow and major bleeding events in Chinese PCI-treated STEMI patients. *Sci. Rep.* **6**, 39412; doi: 10.1038/srep39412 (2016).

**Publisher's note:** Springer Nature remains neutral with regard to jurisdictional claims in published maps and institutional affiliations.

## Supplementary Material

Supplementary Information

## Figures and Tables

**Table 1:** Baseline demographic, clinical, and angiographic characteristics by DPP4a quartiles.

U/L	Total	Q1	Q2	Q3	Q4	P
		≤21.53	21.54–27.14	27.15–32.92	≥32.96	
N	747	187	187	186	187	—
Age (y)	57.84 ± 11.67	59.84 ± 11.50	58.18 ± 11.98	58.01 ± 12.01	55.35 ± 10.78	<0.01
Male, n (%)	618 (82.7)	154 (82.4)	152 (81.3)	158 (84.9)	154 (82.4)	0.81
BMI	25.79 ± 3.40	25.56 ± 3.19	25.76 ± 3.63	25.65 ± 3.22	26.17 ± 3.54	0.32
Hypertension, n (%)	414 (55.4)	115 (61.5)	106 (56.7)	97 (52.2)	96 (51.3)	0.17
Hyperlipidaemia, n (%)	83 (11.1)	25 (13.4)	17 (9.1)	27 (14.6)	14 (7.5)	0.09
Diabetes mellitus, n (%)	165 (22.1)	30 (29.9)	17 (20.3)	27 (21.1)	14 (17.1)	0.02
Current smoker, n (%)	339 (45.4)	77 (48.4)	73 (44.5)	97 (58.4)	92 (54.1)	0.06
Ex-smoker, n (%)	88 (11.8)	28 (25.5)	23 (20.2)	20 (22.5)	17 (17.9)	0.59
Previous MI, n (%)	107 (14.3)	24 (12.8)	21 (11.2)	28 (15.1)	34 (18.2)	0.25
Previous PCI, n (%)	206 (27.6)	53 (28.3)	42 (22.5)	62 (33.5)	49 (26.2)	0.11
Previous CABG, n (%)	7 (0.9)	4 (2.1)	2 (1.2)	0 (0.0)	1 (0.5)	0.17
Medications, n (%)						
Aspirin	742 (99.3)	187 (100.0)	185 (98.9)	184 (98.9)	186 (99.5)	0.53
ACEI/ARB	685 (91.7)	171 (91.4)	173 (92.5)	168 (90.3)	173 (92.5)	0.85
β-blocker	658 (88.1)	166 (88.8)	168 (89.8)	161 (86.6)	163 (87.2)	0.75
Clopidogrel	728 (97.5)	184 (98.4)	185 (98.9)	177 (95.2)	182 (97.3)	0.10
Statin	741 (99.2)	187 (100.0)	185 (98.9)	184 (98.9)	185 (98.9)	0.57
Nitrate	670 (89.7)	172 (92.0)	170 (90.9)	160 (86.0)	168 (89.8)	0.25
Aldosterone antagonist	114 (15.3)	33 (17.6)	26 (13.9)	28 (15.1)	27 (14.4)	0.76
Infarcted location, n (%)						
Anterior	356 (47.7)	89 (47.6)	88 (47.1)	96 (51.6)	83 (44.4)	0.57
Inferior	336 (50.0)	87 (46.6)	83 (44.4)	80 (43.0)	86 (46.0)	0.90
Posterior	32 (4.3)	5 (2.6)	10 (5.3)	7 (3.8)	10 (5.3)	0.51
Lateral	23 (3.1)	6 (3.2)	6 (3.2)	3 (1.6)	8 (4.3)	0.52
Number of disease vessels, n (%)						
Single vessel disease	185 (24.8)	42 (22.5)	51 (27.3)	53 (28.5)	39 (20.9)	0.25
Double vessel disease	147 (19.7)	34 (18.2)	30 (16.0)	38 (20.4)	45 (24.1)	0.24
Triple vessel disease	415 (55.6)	111 (59.3)	106 (56.7)	95 (51.1)	103 (55.0)	0.44
Pre TIMI grade flow, n (%)						
0	457 (61.2)	112 (59.8)	114 (60.9)	112 (60.1)	117 (62.6)	0.95
1	147 (19.7)	42 (22.5)	36 (19.3)	31 (16.7)	38 (20.3)	0.56
2	96 (12.9)	20 (10.7)	26 (13.9)	28 (15.1)	22 (11.8)	0.58
3	49 (6.6)	13 (7)	11 (5.9)	15 (8.1)	10 (5.3)	0.72
Final TIMI grade flow, n (%)						
0	2 (0.3)	0 (0)	0 (0)	1 (0.5)	1 (0.5)	0.57
1	11 (1.5)	1 (0.5)	2 (1.1)	3 (1.6)	5 (2.7)	0.36
2	40 (5.4)	7 (3.7)	9 (4.8)	10 (5.4)	14 (7.5)	0.43
3	694 (92.9)	179 (95.8)	176 (94.1)	172 (92.5)	167 (89.3)	0.09
Myocardial blush grade, n (%)						
0/1	45 (6.0)	6(3.2)	10(5.3)	12(6.5)	17(9.1)	0.12
2	55 (7.4)	9(4.8)	12(6.4)	15(8.1)	19(10.2)	0.11
3	647 (86.6)	172(92.0)	165(88.3)	159(85.4)	151(80.7)	0.01
No reflow phenomenon, n (%)	45 (6.0)	6 (3.2)	10 (5.3)	12 (6.5)	17 (9.1)	0.12
Malignant reperfusion arrhythmia,	37 (5.0)	10 (5.3)	9 (4.6)	7 (3.7)	11 (5.9)	0.81
No. of stent per patients	1.88 ± 1.12	1.50 ± 0.81	1.91 ± 1.22	1.73 ± 1.01	2.36 ± 1.29	0.30
Maximal inflation pressure (atm)	14.28 ± 2.78	14.40 ± 2.27	14.73 ± 3.93	13.82 ± 1.89	14.18 ± 2.89	0.82
Percent diameter stenosis (%)	97.2 ± 3.6	98.1 ± 3.5	94.9 ± 4.4	97.6 ± 3.4	98.0 ± 3.3	0.81
Post percent diameter stenosis (%)	2 ± 1	2 ± 1	2 ± 2	2 ± 1	2 ± 1	0.93
Stent diameter (mm)	3.17 ± 0.41	3.23 ± 0.30	3.25 ± 0.43	3.30 ± 0.49	2.93 ± 0.34	0.14
Total stent length (mm)	50.92 ± 36.09	39.30 ± 18.41	55.27 ± 42.87	43.18 ± 30.82	65.91 ± 43.69	0.36
Thrombus aspiration, n (%)	120 (16.1)	30 (16.0)	26 (13.9)	36 (19.4)	28 (15.0)	0.51
IABP, n (%)	39 (5.2)	9 (4.8)	8 (4.3)	12 (6.5)	10 (5.3)	0.81
CTO, n (%)	19 (2.5)	4 (2.1)	7 (3.7)	3 (1.6)	5 (2.7)	0.60
iMACCE, n (%)	5 (0.7)	1 (0.5)	2 (1.1)	1 (0.5)	1 (0.5)	0.88
IHC, n (%)	91 (12.2)	26 (13.9)	20 (10.7)	23 (12.4)	22 (11.8)	0.82
Major bleeding, n (%)	16 (2.1)	7 (3.7)	4 (2.1)	5 (2.7)	0 (0)	0.08

ACEI, angiotensin-converting-enzyme inhibitor; ARB, angiotensin receptor blckers; BMI, body mass index; CABG, coronary artery bypass grafting; CTO, chronic total occlusion; DPP4a, plasma dipeptidyl peptidase-4 activity; MI, myocardial infarction; IABP, intra-aortic balloon pumping; IHC, in-hospital complications; iMACCE, in-hospital major adverse cardiac or cerebrovascular events; PCI, percutaneous coronary intervention; TIMI, Thrombolysis In Myocardial Infarction.

**Table 2:** Baseline laboratory factors by DPP4a quartiles and their correlations with DPP4a.

U/L	Total	Q1	Q2	Q3	Q4	P	Correlation	
		≤21.53	21.54–27.14	27.15–32.92	≥32.96		r	p
Lipid profile								
Total cholesterol (mg/dL)	4.11 ± 1.04	3.97 ± 1.03	4.06 ± 1.03	4.07 ± 1.08	4.32 ± 1.01	0.01	0.13^a^	<0.01
Triglyceride (mg/dL)	1.56 ± 0.84	1.53 ± 0.71	1.55 ± 0.78	1.45 ± 0.78	1.68 ± 0.85	0.07	0.06^a^	0.11
HDL cholesterol (mg/dL)	1.05 ± 0.29	1.01 ± 0.28	1.03 ± 0.28	1.07 ± 0.29	1.09 ± 0.31	0.06	0.11^a^	0.01
LDL cholesterol (mg/dL)	2.53 ± 0.89	2.42 ± 0.85	2.50 ± 0.85	2.53 ± 0.86	2.67 ± 0.86	0.03	0.11^a^	<0.01
Coagulation profile								
Thrombin time (sec)	16.60 (15.80–18.53)	16.30 (15.60–17.80)	16.70 (15.80–18.20)	16.70 (15.90–19.63)	16.90 (16.20–19.48)	<0.01	0.16^b^	<0.01
Prothrombin time (sec)	13.64 ± 1.40	13.74 ± 1.41	13.62 ± 1.23	13.66 ± 1.08	13.55 ± 1.80	0.67	−0.01^a^	0.83
APTT (sec)	37.85 (34.90–43.33)	38.10 (34.10–41.90)	38.00 (35.28–43.65)	38.05 (35.30–44.43)	37.60 (35.10–42.55)	0.79	0.02^b^	0.76
Fibrinogen (g/L)	3.75 ± 1.16	4.07 ± 1.35	3.91 ± 1.22	3.58 ± 1.01	3.45 ± 0.94	<0.01	−0.21^a^	<0.01
FPB (mmol/l)	6.97 ± 2.71	7.21 ± 2.86	7.00 ± 2.72	6.94 ± 2.90	6.72 ± 2.28	0.53	−0.05^a^	0.20
HbA1C (%)*	6.40 ± 1.38	6.61 ± 1.62	6.20 ± 1.16	6.49 ± 1.47	6.26 ± 1.14	0.13	−0.06^a^	0.27
peak CK-MB (ng/mL)	6.83 (1.83–131.76)	4.52 (1.74–76.99)	7.42 (1.86–162.03)	8.85 (1.88–154.60)	9.85 (1.82–215.43)	0.11	0.10^b^	0.01
peak cTNT (ng/mL)	0.65 (0.45–4.22)	0.62 (0.05–3.21)	0.53 (0.06–3.95)	0.56 (0.30–4.70)	1.08 (0.50–5.26)	0.64	0.05^b^	0.19
peak myoglobin (ng/mL)	49.84 (28.43–384.70)	44.19 (28.03–191.68)	38.09 (29.20–356.58)	49.84 (28.34–618.10)	127.60 (28.00–691.60)	0.12	0.14^b^	<0.01
pro-BNP (pg/mL)	653.90 (250.00–1581.00)	855.70 (250.15–1960.50)	659.20 (241.10–1486.00)	687.90 (282.70–1611.00)	553.70 (234.23–1396.00)	0.21	−0.07^b^	0.06
Creatinine (umol/L)	78.30 (68.20–90.18)	79.70 (65.30–95.80)	79.90 (66.45–91.75)	78.50 (70.28–91.65)	76.35 (67.45–85.50)	0.09	−0.07^b^	0.049
GGT (U/L)	32.80 (21.70–53.90)	30.40 (21.33–45.78)	30.60 (21.20–54.30)	31.00 (22.60–48.95)	38.00 (23.60–73.10)	<0.01	0.14^b^	<0.01

APTT, activated partial thromboplastin time; BNP, brain natriuretic peptide; CK-MB, MB isoenzyme of creatine kinase; cTNT, cardiac troponin T; DPP4a, plasma dipeptidyl peptidase-4 activity; FPG, fasting plasma glucose; GGT, gamma-glutamyltransferase; HbA1c, hemoglobin A1c; HDL, high-density lipoprotein; LDL, low-density lipoprotein;

^a^Pearson correlation coefficients; ^b^Spearman correlation coefficients.

^*^HbA1c was only detected in 373 patients; Number of patients detected were 107 in Q1, 88 in Q2, 88 in Q3 and 89 in Q4.

**Table 3:** Associations between various demographic and laboratory factors and DPP4a.

	Β	95% CI	p
Age	−0.10	(−0.18, −0.02)	0.01
Fibrinogen	−1.27	(−2.12, −0.43)	<0.01
Peak CK-MB	0.01	(0.006, 0.02)	<0.01
GGT	0.04	(0.02, 0.06)	<0.01

B-coefficients and 95% CI meant the unstandardized coefficients and their 95% confidence interval. The following variables were removed from the model: gender, body mass index (BMI), total cholesterol, low-density lipoprotein (LDL) cholesterol, high-density lipoprotein (HDL) cholesterol, triglycerides, peak myoglobin, peak cardiac troponin (cTNT), fasting plasma glucose, thrombin time, prothrombin time, activated partial thromboplastin time (APTT), D-dimers, pro-brain natriuretic peptide (pro-BNP), creatinine, history of hypertension, previous myocardial infarction (MI), smoking status, use of an angiotensin-converting-enzyme inhibitor (ACEI) or an angiotensin receptor blocker (ARB), statin use, aspirin use, β-blocker use, and aldosterone antagonist use.

CK-MB, MB isoenzyme of creatine kinase; DPP4a, plasma dipeptidyl peptidase-4 activity; GGT, gamma-glutamyltransferase.

**Table 4:** Associations between DPP4a and no-reflow events, iMACCE, IHC, and in-hospital major bleeding events.

	Unadjusted	p Value	Model 1*	p Value	Model 2†	p
	OR (95% CI)		OR (95% CI)		OR (95% CI)	
No reflow phenomenon						
Q1	1.00 (reference)		1.00 (reference)	1.00 (reference)		
Q2	1.70 (0.61–4.79)	0.3	1.71 (0.61–4.82)	0.31	1.38 (0.41–4.63)	0.61
Q3	2.08 (0.76–5.67)	0.12	2.16 (0.79–5.91)	0.13	2.81 (0.89–8.85)	0.08
Q4	3.02 (1.16–7.83)	0.02	3.16 (1.20–8.29)	0.02	3.39 (1.08–10.61)	0.04
per 1 U/L	1.05 (1.02–1.09)	<0.01	1.06 (1.02–1.09)	<0.01	1.07 (1.02–1.11)	<0.01
iMACCE						
Q1	1.00 (reference)		1.00 (reference)	1.00 (reference)		
Q2	1.00 (0.14–7.17)	1.00	1.00 (0.14–7.28)	1.00	0.88 (0.11–7.23)	0.91
Q3	0.50 (0.04–5.56)	0.57	0.48 (0.04–5.38)	0.55	0.50 (0.04–6.13)	0.59
Q4	0.50 (0.05–5.53)	0.57	0.49 (0.04–5.58)	0.56	0.45 (0.04–5.91)	0.55
per 1 U/L	0.97 (0.88–1.07)	0.52	0.97 (0.88–1.07)	0.51	0.97 (0.88–1.07)	0.57
IHC						
Q1	1.00 (reference)		1.00 (reference)	1.00 (reference)		
Q2	0.74 (0.40–1.38)	0.35	0.78 (0.41–1.45)	0.43	0.99 (0.47–2.09)	0.98
Q3	0.87 (0.48–1.60)	0.66	0.92 (0.50–1.68)	0.78	1.03 (0.48–2.18)	0.95
Q4	0.83 (0.45–1.52)	0.54	0.95 (0.51–1.76)	0.87	0.90(0.40–2.03)	0.81
per 1 U/L	1.00 (0.98–1.03)	0.86	1.01 (0.98–1.04)	0.53	1.01 (0.98–1.04)	0.40
Major bleeding						
Q1	1.00 (reference)		1.00 (reference)	1.00 (reference)		
Q2	0.56 (0.16–1.95)	0.36	0.57 (0.17–2.00)	0.38	0.61 (0.10–3.75)	0.54
Q3	0.71 (0.22–2.28)	0.57	0.74 (0.23–2.39)	0.62	1.04 (0.22–5.01)	0.95
Q4	—		—		—	
per 1 U/L	0.92 (0.86–0.98)	0.01	0.92 (0.86–0.98)	0.01	0.90 (0.82–0.98)	0.02

IHC, in-hospital complications; iMACCE, in-hospital major adverse cardiac or cerebrovascular events; DPP4a, plasma dipeptidyl peptidase-4 activity; OR, odds ratio; CI, confidence interval.

^*^Adjusted for age and sex.

^†^Adjusted for age, sex, BMI, creatinine, GGT, total cholesterol, fibrinogen, peak CK-MB, pro-BNP, fasting plasma glucose, hypertension, previous MI, smoking status, ACEI/ARB use, and β-blocker use.
