# Supplementary material for: Plasma DPP4 activity is associated with no-reflow and major bleeding events in Chinese PCI-treated STEMI patients
Source: Sci Rep. 2016 Dec 21;6:39412. doi: 10.1038/srep39412 (PMC5175165; doi:10.1038/srep39412)
Supplement: Supplementary Information [file srep39412-s1.pdf]

Plasma DPP4 activity is associated with no-reflow and major bleeding events in Chinese PCI-treated STEMI patients

Jing Wei Li* M.D., Yun Dai Chen† M.D., Wei Ren Chen* M.D., Jing Jing M.D., Jie Liu M.D., Yong Qiang Yang M.D.

Department of Cardiology, People's Liberation Army General Hospital, Beijing, China

*These authors contributed equally to this work.

†Corresponding author:

Yun Dai Chen M.D. Tel. /fax: +8610-66939709.

E-mail address: cydplagh@sina.com

Address for correspondence: No.28 Fuxing Road, Wukesong, Haidian District, Beijing, China, 100853

Total word count: 3161

Brief title: DPP4 activity and in-hospital events after PCI

Supp. Table 1. Correlations of DPP4a with age and laboratory factors in NSTEMI patients

	Spearman r	p
Age	-0.15[a]	0.08
Total cholesterol (mg/dL)	0.18[a]	0.04
Triglyceride (mg/dL)	-0.10[a]	0.27
HDL cholesterol (mg/dL)	0.14[a]	0.10
LDL cholesterol (mg/dL)	0.21[a]	0.02
Thrombin time (sec)	0.02	0.84
Prothrombin time (sec)	0.08[a]	0.39
APTT (sec)	0.06	0.65
Fibrinogen (g/L)	-0.09[a]	0.30
Plasma glucose mmol/l	0.05[a]	0.59
HbA1C (%)	0.03[a]	0.82
Peak CK-MB (ng/mL)	0.14	0.09
Peak cTNT (ng/mL)	0.15	0.08
Peak myoglobin (ng/mL)	-0.01	0.93
Pro-BNP (pg/mL)	-0.02	0.85

Creatinine (umol/L)	-0.06	0.51
GGT (U/L)	0.15	0.09

[a]Pearson’s correlation analysis

APTT, activated partial thromboplastin time; HbA1c, hemoglobin A1c; CK-MB, MB isoenzyme of creatine kinase; cTNT, cardiac troponin T; BNP, brain natriuretic peptide; GGT, gamma-glutamyl transferase
